# Author’s reply to “Methodological considerations for nonconcurrent comparisons of pulsed field ablation catheter platforms”

**DOI:** 10.1016/j.hroo.2026.01.005

**Published:** 2026-01-12

**Authors:** Joerg Yogarajah, Julie Hutter, Patrick Kahle, Marko Tomic, Mirlinda Lüsebrink, Andreas Hain, Samuel Sossalla, Malte Kuniss, Thomas Neumann

**Affiliations:** 1 Department of Cardiology, Kerckhoff Heart Center, Campus Kerckhoff, Justus Liebig University Giessen, Bad Nauheim, Germany; 2 Department of Cardiology, Medical Clinic I, Justus Liebig University Giessen, Giessen, Germany

We thank the authors for their constructive comments and the opportunity to clarify methodological aspects of our study.^1^

We agree that the nonrandomized, nonconcurrent design limits causal inference. Catheter choice was largely driven by calendar time during early implementation, making the analysis susceptible to secular trends and workflow evolution. A contemporaneous comparison could have biased results toward the fixed-loop circular catheter (FLCC), as operators had prior experience, whereas the variable-loop circular catheter (VLCC) was new. Analyzing the first 45 consecutive cases of each catheter provides a pragmatic view of real-world adoption and acute performance. Similar studies using different time periods have been published with other pulsed field ablation systems.^2^

Baseline characteristics differed, but were unlikely to affect acute procedural outcomes, including pulmonary vein isolation success. Differences may influence long-term arrhythmia recurrence, representing a limitation for extended outcomes, but are unlikely to substantially affect acute efficacy or safety.

Adjunctive ablation procedures were described descriptively. Statistical comparisons of application numbers were not performed because of unequal group distributions and differing indications; these data illustrate VLCC versatility beyond pulmonary vein isolation compared to FLCC rather than formal hypothesis testing.

Procedural guidance differed intentionally: fluoroscopy-only for FLCC vs 3-dimensional electroanatomic mapping for VLCC, reflecting real-world workflows. Observed differences in procedure time and fluoroscopy exposure represent system-specific characteristics rather than uncontrolled bias. As with cryoballoon vs radiofrequency catheters, differences in guidance (fluoroscopy vs 3-dimensional) account for workflow variations and reflect system-specific features, not bias.^3^

In summary, our findings provide early real-world evidence on feasibility, workflow differences, acute safety, and novel applications of circular pulsed field ablation systems during the clinical implementation phase. Furthermore, larger prospective studies with standardized protocols and long-term follow-up are warranted to formally compare catheter platforms and assess lesion durability and clinical outcomes.
